# Simultaneous Assessment of Asp Isomerization and Asn Deamidation in Recombinant Antibodies by LC-MS following Incubation at Elevated Temperatures

**DOI:** 10.1371/journal.pone.0030295

**Published:** 2012-01-17

**Authors:** Katharina Diepold, Katrin Bomans, Michael Wiedmann, Boris Zimmermann, Andreas Petzold, Tilman Schlothauer, Robert Mueller, Bernd Moritz, Jan Olaf Stracke, Michael Mølhøj, Dietmar Reusch, Patrick Bulau

**Affiliations:** 1 Pharma Research and Development Penzberg, Roche Diagnostics GmbH, Penzberg, Germany; 2 Pharma Biotech Penzberg, Roche Diagnostics GmbH, Penzberg, Germany; 3 Late-Stage Pharmaceutical and Processing Development, F. Hoffmann-La Roche Ltd., Basel, Switzerland; 4 Analytical Research and Development, F. Hoffmann-La Roche Ltd., Basel, Switzerland; University of Giessen Lung Center, Germany

## Abstract

The degradation of proteins by asparagine deamidation and aspartate isomerization is one of several chemical degradation pathways for recombinant antibodies. In this study, we have identified two solvent accessible degradation sites (light chain aspartate-56 and heavy chain aspartate-99/101) in the complementary-determining regions of a recombinant IgG1 antibody susceptible to isomerization under elevated temperature conditions. For both hot-spots, the degree of isomerization was found to be significantly higher than the deamidation of asparagine-(387, 392, 393) in the conserved CH3 region, which has been identified as being solvent accessible and sensitive to chemical degradation in previous studies. In order to reduce the time for simultaneous identification and functional evaluation of potential asparagine deamidation and aspartate isomerization sites, a test system employing accelerated temperature conditions and proteolytic peptide mapping combined with quantitative UPLC-MS was developed. This method occupies the formulation buffer system histidine/HCl (20 mM; pH 6.0) for denaturation/reduction/digestion and eliminates the alkylation step. The achieved degree of asparagine deamidation and aspartate isomerization was adequate to identify the functional consequence by binding studies. In summary, the here presented approach greatly facilitates the evaluation of fermentation, purification, formulation, and storage conditions on antibody asparagine deamidation and aspartate isomerization by monitoring susceptible marker peptides located in the complementary-determining regions of recombinant antibodies.

## Introduction

Degradation of proteins by asparagine (Asn) deamidation and aspartate (Asp) isomerization has been extensively reviewed [1], [2], [3], [4], [5], [6]. Previous studies have identified that degradation of Asn and Asp residues in proteins can impact *in vivo* biological functions and *in vitro* stability [7], [8], [9], [10], [11]. Recombinant monoclonal antibodies (mAbs) are exposed to process and storage conditions that might influence the rate of deamidation and isomerization. Cacia *et al.* demonstrated that the light chain Asp- 32 of an anti-IgE antibody could be converted to a succinimide intermediate (Asu) and iso-aspartate (iso-Asp) [9]. Three IgG1 mAbs have been reported to lose activity because of deamidation or isomerization in the complementary-determining regions (CDRs) of the heavy chain [12], [13], [14]. In case of the recombinant IgG1 antibody Herceptin (HER2), the loss of potency was caused by the isomerization of heavy chain Asp-102 (CDR 3). Deamidation of the light chain Asn-30 (CDR 1) did not significantly impact the HER2 potency [12]. Two independent studies reported the heavy chain Asn-55 (CDR 2) to be susceptible to deamidation *in vivo* [13] and to exist in a stable succinimide form at mildly acidic pH [14]. In another investigation, the light chain Asp-30 of an IgG2 antibody was found to form succinimide and iso-Asp. However, no significant impact on the biological function was reported [15]. Chelius *et al.* applied accelerated degradation conditions to identify four potential deamidation sites in the conserved regions of recombinant IgG1 monoclonal antibodies [16].

Analytical characterization of Asn deamidation and Asp isomerization has been conducted by enzymatic methylation [17], [18], chemical hydrolysis with hydroxylamine [19], by chromatographic fractionation followed by Edman sequencing, and tryptic digestions [9], [15], [20]. Approaches involving mass spectrometry provide alternative solutions for the characterization of deamidation and isomerization events. Advances in high resolution mass spectrometry has enabled the analysis of Asn deamidation in intact proteins [21], [22], [23], [24]. However, for large biomolecules such as recombinant antibodies, bottom-up liquid chromatography-mass spectrometry (LC-MS) of proteolytic peptides is often the method of choice for monitoring site specific deamidation or isomerization reactions [13], [14], [15], [16], [25], [26], [27], [28], [29].

In the present study, an approach employing elevated temperatures and proteolytic peptide mapping combined with quantitative LC-MS for the simultaneous induction, identification and quantification of Asn deamidation and Asp isomerization in recombinant antibodies was developed. This test system allowed us to identify light chain Asp-56 (CDR 2) and heavy chain Asp-99/101 (CDR 3) as potential sites for Asp isomerization in recombinant IgG1 antibodies.

## Results

**Figure 1:**
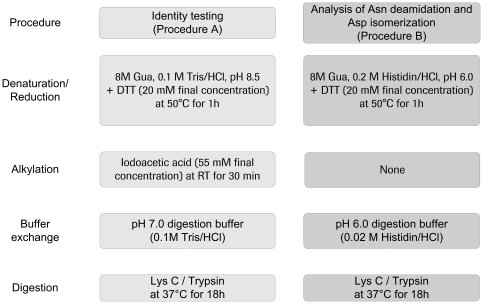
Overview of sample preparation procedures A and B for the UPLC-MS analysis of antibody Asn deamidation and Asp isomerization.

The developed test system for the simultaneous identification and quantification of Asn deamidation and Asp isomerization in recombinant antibodies employs proteolytic peptide mapping at mildly acid conditions combined with quantitative UPLC-MS. In general, the new method (procedure B) involves denaturation and reduction at pH 6.0 leaving out any alkylation step, followed by proteolytic digestion also at pH 6.0 in histidine/HCl (Figure 1). To demonstrate that the developed sample preparation procedure significantly reduces method-induced Asn deamidation, reference material (stored at −80°C) of the recombinant IgG1 antibody HER2 was analyzed according to the sample preparation procedures A and B depicted in Figure 1. The extent of quantifiable Asn deamidation and Asp isomerization was determined by quantitative evaluation of specific ion current chromatograms of modified LysC peptides and their unmodified parent peptides using the quantification software GRAMS/32 (Table 1). For all light or heavy chain Asn residues the application of the procedure B resulted in a significant reduction of method-induced deamidation events (Table 1; 29% up to 94%). In addition, the Asu content was stabilized for all monitored Asn and Asp residues as a consequence of the mild acid conditions (Tables 1, 2, 3). Moreover, the HER2 LysC peptide containing the HC-iso-Asp-102 was only chromatographically resolved after applying procedure B.

**Table 1:** Comparison of the pH 7.0 (procedure A) and pH 6.0 (procedure B) sample preparation procedures for the UPLC-MS analysis of HER2 Asn deamidation and Asp isomerizations.

	Procedure A	Procedure B
LC-**Asn**-30		
LC-Asu-30	n.d.	2.6
LC-Asp-30	1.2	0.4
LC-iso-Asp-30	13.5	7.5
HC-**Asn**-28		
HC-Asu-28	n.d.	n.d.
HC-deamid-28	5.0	n.d.
HC-**Asn**-55		
HC-Asu-55	4.7	5.6
HC-deamid-55	29.9	3.6
HC-**Asp**-102		
HC-Asu-102	1.8	2.1
HC-iso-Asp-102	n.q.	2.5
HC-**Asp**-283		
HC-Asu-283	4.1	4.8
HC-iso-Asp-283	1.6	0.9
HC-**Asn**-364		
HC-Asu-364	0.2	0.1
HC-deamid-364	7.4	1.7
HC-**Asn**-387,392,393		
HC-Asu-387,392,393	6.3	0.7
HC-deamid-387,392,393	9.8	0.3
HC-**Asp**-404		
HC-Asu-404	2.2	3.8
HC-iso-Asp-404	n.d.	n.d.
HC-**Asn**-424,437		
HC-Asu-424,437	n.d.	n.d.
HC-deamid-424,437	12.9	8.0

Relative quantification (in %) was conducted by specific ion current chromatogram analysis of proteolytic LysC peptides using the quantification software GRAMS/32™. deamid, total Asp/iso-Asp; n.d., not detectable; n.q., not quantifiable.

**Table 2:** Assessment of HER2 Asn deamidation and Asp isomerization using accelerated degradation conditions and quantitative UPLC-MS (Procedure B).

Storage Duration/Temperature	RM/80°C	1 M/25°C	2 M/25°C	7 d/40°C	1 M/40°C	2 M/40°C
LC-**Asn**-30						
LC-Asu-30	2.6 (±0.2)	9.0 (±0.3)	11.0 (±0.8)	12.5 (±0.3)	23.4 (±1.8)	24.8 (±1.7)
LC-Asp-30	0.4 (±0.1)	2.1 (±0.1)	3.1 (±0.1)	3.2 (±0.2)	4.7 (±0.1)	7.0 (±0.6)
LC-isoAsp-30	7.5 (±0.7)	10.9 (±0.4)	15.3 (±1.1)	11.1 (±1.1)	18.8 (±2.6)	26.5 (±0.2)
HC-**Asn**-55						
HC-Asu-55	5.6 (±0.4)	5.5 (±0.7)	5.1 (±0.9)	5.5 (±0.4)	5.2 (±0.4)	5.4 (±0.6)
HC-deamid-55	3.6 (±0.4)	4.1 (±0.1)	3.8 (±0.6)	4.3 (±0.1)	5.4 (±0.5)	8.9 (±1.1)
HC-**Asp**-102						
HC-Asu-102	2.1 (±0.2)	2.8 (±0.1)	2.8 (±0.1)	3.4 (±0.1)	3.1 (±0.4)	3.8 (±0.1)
HC-isoAsp-102	2.5 (±0.4)	9.0 (±0.5)	15.9 (±0.6)	14.6 (±0.2)	25.9 (±1.2)	46.1 (±1.0)
HC-**Asp**-283						
HC-Asu-283	4.8 (±0.6)	5.2 (±0.7)	5.7 (±1.0)	5.9 (±1.3)	5.8 (±0.1)	5.6 (±0.5)
HC-isoAsp-283	0.9 (±0.1)	1.0 (±0.1)	1.3 (±0.3)	1.3 (±0.2)	2.1 (±0.1)	3.4 (±0.1)
HC-**Asn**-387,392,393						
HC-Asu-387,392,393	0.7 (±0.1)	0.7 (±0.1)	1.0 (±0.1)	0.8 (±0.1)	0.9 (±0.1)	0.9 (±0.1)
HC-deamid-387,392,393	0.3 (±0.1)	0.8 (±0.1)	1.4 (±0.4)	1.0 (±0.1)	1.8 (±0.1)	3.8 (±0.1)
**CEC**						
% Acidic	27.7	32.7	39.8	33.7	46.7	n.q.
% Main	63.5	51.3	38.5	45.0	26.0	n.q
% Basic	8.8	16.0	21.6	21.3	27.3	n.q
**SEC**						
% Fragment	<0.1	0.1	0.2	0.2	0.4	1.0
% Monomer	99.8	99.6	99.4	99.4	99.0	98.2
% Aggregate	0.2	0.3	0.4	0.4	0.6	0.8
**SPR**						
% Target Binding	100 (±2)	93 (±2)	89 (±2)	90 (±2)	79 (±2)	59 (±3)

Relative quantification (in %) was conducted by specific ion current chromatogram analysis of proteolytic LysC peptides using the quantification software GRAMS/32™ (n = 2, mean ± S.D). HER2 charge variants were monitored by cation-exchange chromatography (CEC). Formation of fragments and aggregates was monitored by size-exclusion chromatography (SEC) and target binding activity was assessed by SPR-analysis. deamid, total Asp/iso-Asp; n.q., not quantifiable; RM, Reference material.

**Table 3:** Identification and evaluation of Mab1 Asn deamidation and Asp isomerization sites using accelerated degradation conditions and quantitative UPLC-MS (Procedure B).

Storage Duration/Temperature	RM/80°C	1 M/25°C	2 M/25°C	7 d/40°C	1 M/40°C	2 M/40°C
LC-**Asn**-30						
LC-Asu-30	n.q.	n.q.	n.q.	n.q.	n.q.	n.q.
LC-deamid-30	n.q.	n.q.	n.q.	n.q.	n.q.	n.q.
**(LysC digestion)**						
LC-**Asp**-56						
LC-Asu-56	n.q.	n.q.	n.q.	n.q.	n.q.	n.q.
LC-isoAsp-56	n.q.	n.q.	n.q.	n.q.	n.q.	n.q.
(**LysC digestion**)						
HC-**Asp**-99/101						
HC-Asu-99/101	9.1 (±1.1)	16.2 (±1.4)	19.8 (±1.3)	19.9 (±0.5)	32.0 (±0.6)	38.2 (±0.4)
HC-isoAsp-99/101	n.q.	n.q.	n.q.	n.q.	n.q.	n.q.
(**LysC digestion**)						
LC-**Asn**-30						
LC-Asu-30	0.3 (±0.1)	0.3 (±0.1)	0.4 (±0.1)	0.5 (±0.1)	0.7 (±0.1)	0.9 (±0.1)
LC-deamid-30	3.4 (±1.4)	3.1 (±0.9)	3.2 (±1.0)	3.1 (±1.5)	3.4 (±2.0)	4.2 (±1.4)
(**Trypsin digestion**)						
LC-**Asp**-56						
LC-Asu-56	6.2 (±0.8)	8.5 (±1.1)	9.4 (±1.5)	9.5 (±0.7)	11.9 (±0.3)	12.6 (±0.6)
LC-isoAsp-56	2.3 (±0.4)	3.9 (±1.1)	5.5 (±1.3)	4.5 (±1.2)	9.0 (±1.2)	11.3 (±1.3)
(**Trypsin digestion**)						
HC-**Asp**-99/101						
HC-Asu-99/101	2.9 (±0.1)	4.7 (±0.4)	5.1 (±0.4)	4.9 (±0.6)	6.0 (±0.6)	6.7 (±0.6)
HC-isoAsp-99/101	9.4 (±0.1)	17.6 (±0.3)	22.1 (±0.1)	21.4 (±0.1)	40.0 (±0.3)	55.6 (±0.8)
(**Trypsin digestion**)						
HC-**Asn**-389,394,395						
HC-Asu-389,394,395	0.8 (±0.1)	0.8 (±0.1)	0.8 (±0.1)	0.9 (±0.1)	0.9 (±0.1)	0.9 (±0.1)
HC-deamid-389,394,395	2.1 (±0.2)	2.1 (±0.2)	2.3 (±0.1)	3.2 (±0.1)	3.2 (±0.1)	4.3 (±0.1)
(**LysC digestion**)						
**CEC**						
% Acidic	18.6	17.1	19.1	16.4	17.6	22.7
% Main	59.7	45.3	36.9	37.7	21.1	17.3
% Basic	21.7	37.6	44.0	45.9	61.3	60.0
**SEC**						
% Fragment	<0.1	<0.3	0.3	<0.3	0.5	5.4
% Monomer	98.7	98.5	98.4	98.6	98.1	92.7
% Aggregate	1.3	1.3	1.3	1.2	1.4	1.9
**SPR**						
% Target Binding	100 (±2)	90 (±2)	83 (±1)	85 (±2)	53 (±2)	22 (±1)

Relative quantification (in %) was conducted by specific ion current chromatogram analysis of proteolytic peptides (LysC or trypsin) using the quantification software GRAMS/32™ (n = 2, mean ± S.D). MAB1 charge variants were monitored by cation-exchange chromatography (CEC). Formation of fragments and aggregates was monitored by size-exclusion chromatography (SEC) and target binding activity was assessed by SPR-analysis. deamid, total Asp/iso-Asp; n.q., not quantifiable; RM, Reference material.

**Figure 2:**
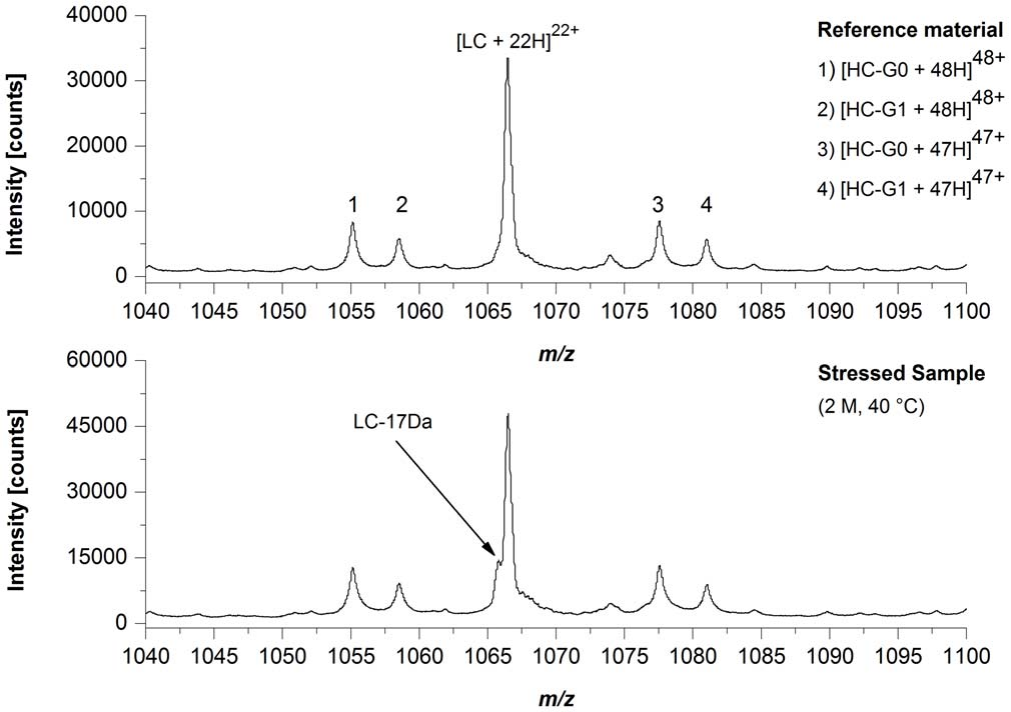
ESI-QTOF mass spectrometry of reduced HER2. NanoESI-QTOF mass spectra of HER2 reference material and HER2 stressed sample (stored at 40°C for 2 month). The spectra were recorded in the positive ion mode using acetonitrile/water/formic acid (78/20/2, v/v/v) as solvent. LC, light chain; HC-G0, non-galactosylated heavy chain; HC-G1, mono-galactosylated heavy chain.

To assess potential sites for HER2 degradation, we exposed HER2 reference material to elevated temperature conditions (25°C and 40°C) for up to 2 month followed by LysC peptide mapping according to procedure B combined with quantitative UPLC-MS. Table 2 summarizes the quantification results for those HER2 amino acid residues that showed significant alterations in Asn deamidation or Asp isomerization. For LC-Asn-30 (located in the CDR 1) we found significantly increased levels of Asu, Asp, and iso-Asp at both temperatures. The appearance of around 25% LC-Asu after 2 months at 40°C was further demonstrated by off-line electrospray ionization mass spectrometry (ESI-MS) of reduced HER2 material where a loss of 17 Da was only observed for the antibody LC of stressed HER2 material (Figure 2). In contrast, the Asu level of HC-Asn-55 (located in the CDR 2) was not affected by the elevated temperatures and only a moderate increase in deamidation (3.6% to 8.9%) was observed at 40°C after 2 month (Table 2). The HC-Asp-102 (located in the CDR 3) displayed a demonstrative elevation in iso-Asp formation upon application of temperature stress (from 2.5% up to 46.1% after 2 M incubation at 40°C); however, only a minimal increase of the HC-Asu-102 level was detected (Table 2). For the HC-Asp-283 and HC-Asn-(387, 392, 393), both located in the conserved region of recombinant IgG1 antibodies, no significant changes in Asu formation, but a moderate increase of iso-Asp and Asp/iso-Asp formation, respectively, was observed following incubation at 40°C. Consequently, the significant alterations in Asn deamidation and Asp isomerization upon temperature stress resulted in significant changes of HER2 charge variants (determined by cation-exchange chromatography, Table 2). However, the preservation of structural integrity was verified by size-exclusion chromatography analysis where only a minimal increase of fragment or aggregate formation could be demonstrated (Table 2). Moreover, the increase of Asn deamidation and Asp isomerization lead to a significant loss of target binding activity. A previous study has already identified LC-Asn-30 and HC-Asp-102 as potential sites for HER2 degradation and the criticality of HC-iso-Asp-102 formation for HER2 potency was demonstrated [12].

Thus, the application of elevated temperatures combined with quantitative UPLC-MS and binding studies was adequate to assess potential sites for HER2 Asn deamidation and Asp isomerization.

**Figure 3:**
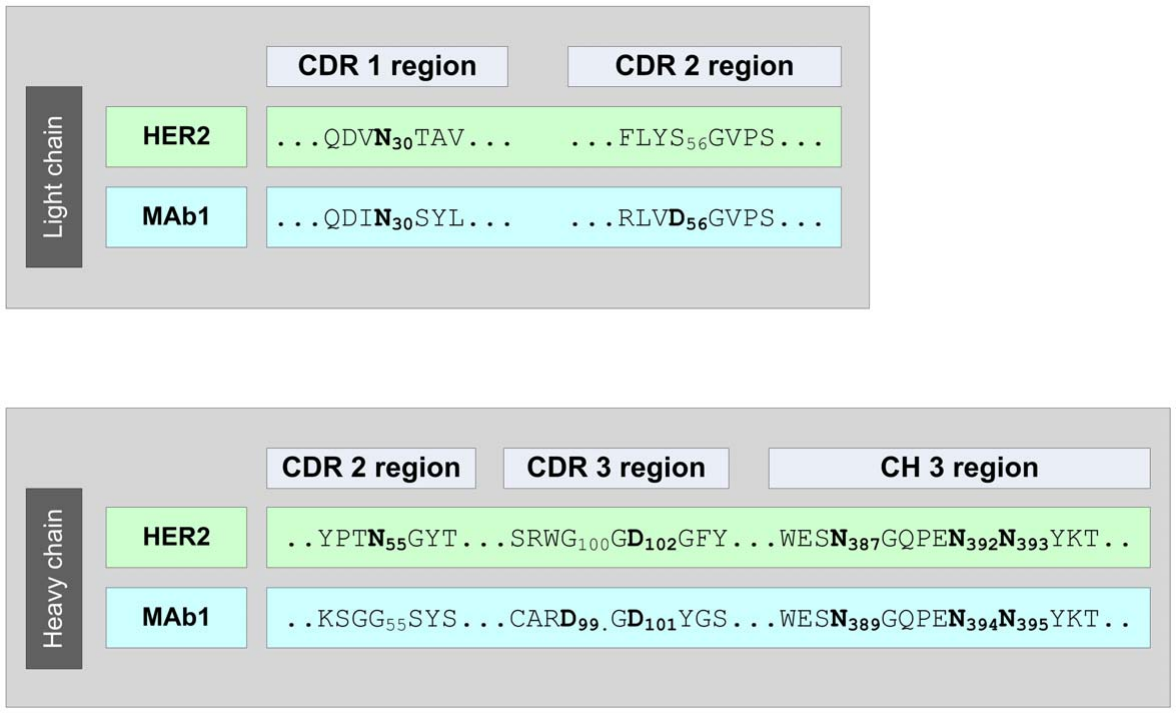
Partial sequences of HER2 and Mab1.

**Figure 4:**
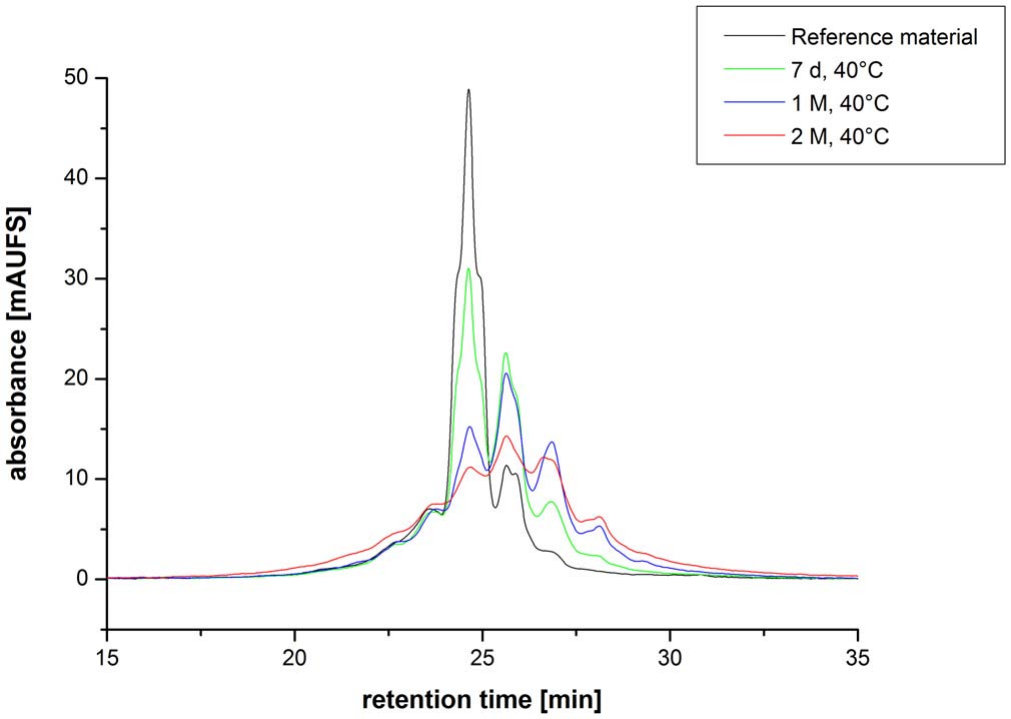
Cation-exchange chromatography of Mab1 reference material versus selected stressed samples (stored at 40°C; 7 days, 1 and 2 month).

**Figure 5:**
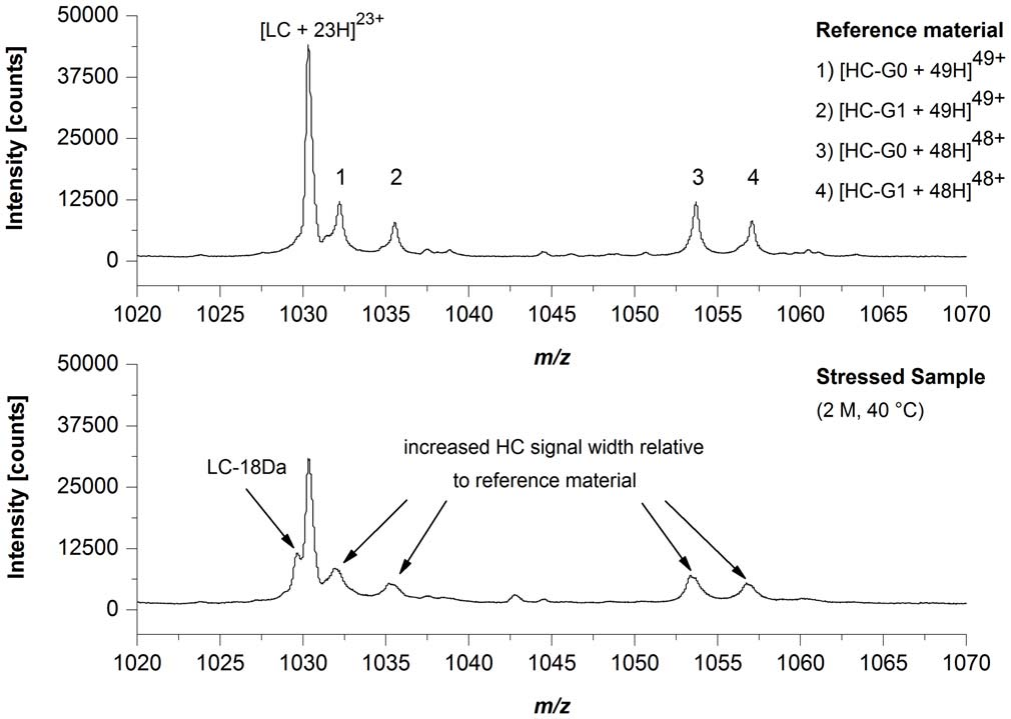
ESI-QTOF mass spectrometry of reduced Mab1. NanoESI-QTOF mass spectra of Mab1 reference material and Mab1 stressed sample (stored at 40°C for 2 month). The spectra were recorded in the positive ion mode using acetonitrile/water/formic acid (79/20/1, v/v/v) as solvent. LC, light chain; HC-G0, non-galactosylated heavy chain; HC-G1, mono-galactosylated heavy chain.

**Figure 6:**
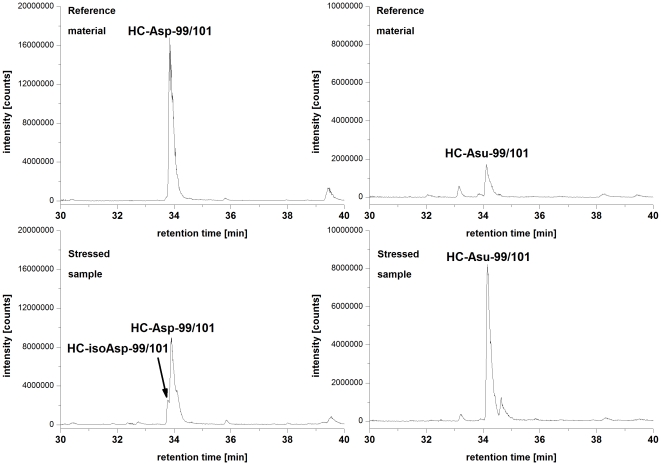
Specific ion chromatograms (SICs) of Mab1 peptides containing LC-Asx-99/101. M = 5464.46 Da *(z = 3, 4, and 5)* (HC-Asp-99/101; HC-iso-Asp-99/101) and M = 5446.45 Da *(z = 3, 4, and 5)* (HC-Asu-99/101) from the Mab1 reference material and a stressed sample (stored at 40°C for 2 month).

**Figure 7:**
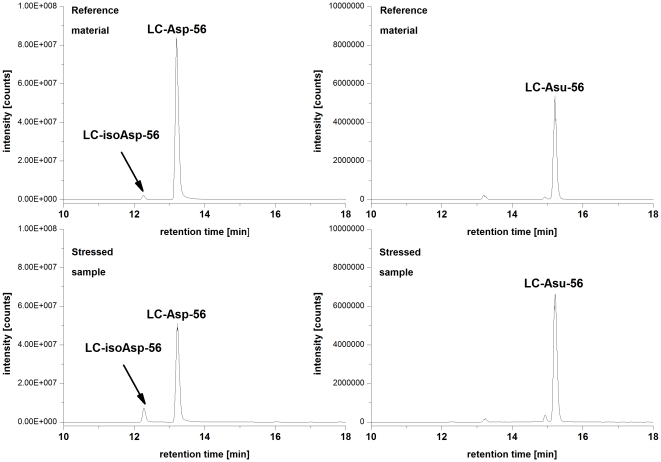
Specific ion chromatograms (SICs) of Mab1 peptides containing LC-Asx-56. M = 841.47 Da *(z = 1, and 2)* (LC-Asp-56; LC-iso-Asp-56) and M = 823.46 Da *(z = 1, and 2)* (LC-Asu-56) from the Mab1 reference material and a stressed sample (stored at 40°C for 2 month).

Next, we employed the described approach to identify critical quality attributes (CQAs) for an IgG1 in early clinical development (Mab1). Mab1 shares with HER2 the LC-Asn-30 in the CDR 1 and offers a HC-Asp-99/Gly-100/Asp-101 motive in the CDR 3. Moreover, Mab1 exhibits a novel LC-Asp-56/Gly-57 motive in the CDR 2 (Figure 3). Following incubation of Mab1 at elevated temperatures, a significant increase of basic charge variants were observed, whereas only a slight increase of acidic charge variants could be detected (Figure 4, Table 3). In addition, ESI-MS analysis of reduced Mab1 material indicated a loss H_2_O (−18 Da) for the antibody light and heavy chain (Figure 5) of stressed Mab1 material. In conclusion, the data suggest a significant formation of Asu and only a minor formation of Asp. Next, the stressed samples were analyzed by LysC peptide mapping combined with quantitative UPLC-MS. The LysC peptide mapping approach resulted in the identification of distinct Asu and moderate iso-Asp formation at HC-Asp-99/Asp-101 (Figure 6, Table 3). In addition, a moderate elevation of deamidation at the conserved HC-Asn-(389, 394, 395) was observed (Table 3). However, due to inadequate chromatographic separation of the generated LysC peptides it was not possible to relatively quantify the amount of deamidation at LC-Asn-30 and the isomerization at LC-Asp-56. Therefore, procedure B and tryptic peptide mapping was applied to generate shorter Mab1 peptides. The tryptic peptide mapping approach demonstrated minimal deamidation at LC-Asn-30 but significant Asu and iso-Asp formation at LC-Asp-56 (Figure 7, Table 3). Moreover, massive formation of iso-Asp was observed at HC-Asp-99/Asp-101 which is in contrast to the results from ESI-MS analysis and LysC peptide mapping which both suggest the primary formation of Asu instead of iso-Asp (Figure 5, Table 3). This observation is most likely due to the *N*-terminal location of the HC-Asp-99/Gly-100/Asp-101 motive at tryptic peptide level (see tryptic cleavage site HC-Arg-98 in Figure 3) which likely promotes the conversion of Asu to iso-Asp. Thus, the demonstrative iso-Asp formation at HC-Asp-99/Asp-101 of the tryptic peptide is regarded a method artifact.

**Figure 8:**
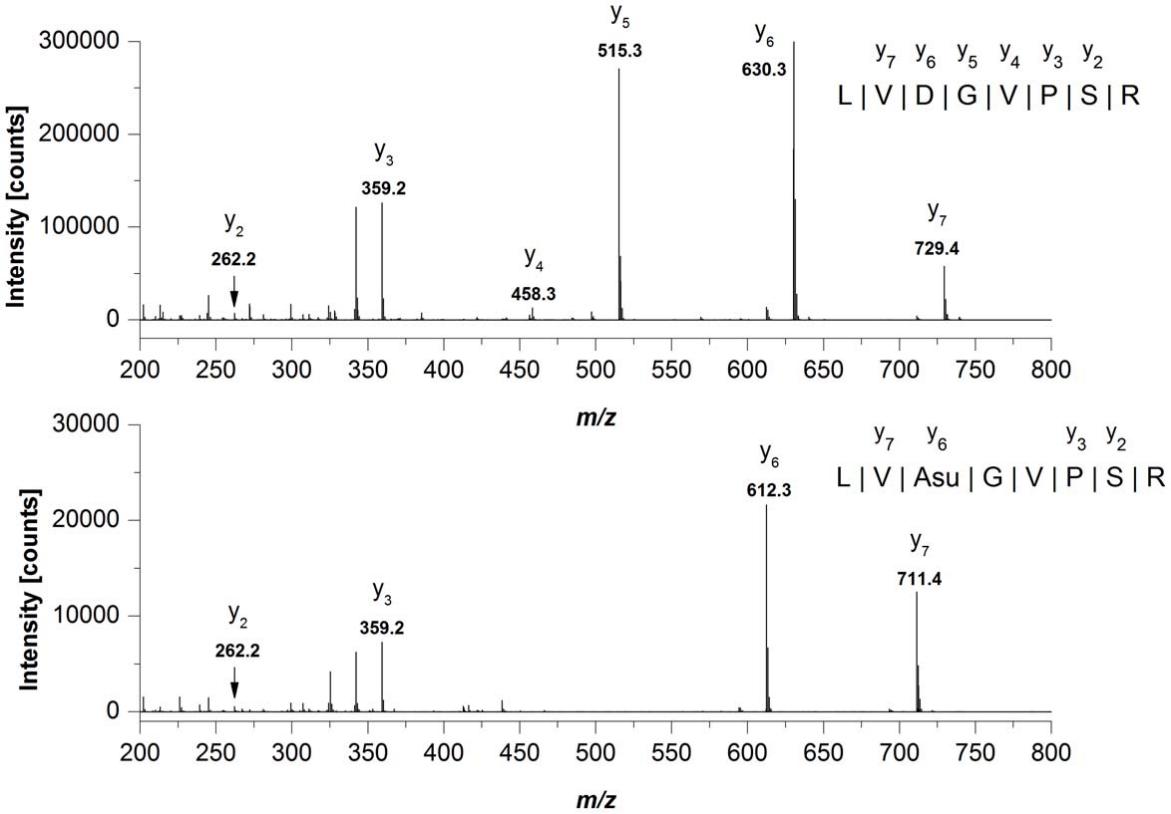
Low-energy CID mass spectra and resulting amino acid sequence of the doubly protonated Mab1 light chain peptide containing Asp-56 (upper panel) and Asu-56 (lower panel) at *m/z* 421.7 and 412.7.

Sequence determination of all LysC/tryptic Mab1 peptides selected for quantitative evaluation of deamidation and isomerization events was carried out on-line by low-energy CID (exemplarily shown for the tryptic peptide containing LC-Asu-56, Figure 8). Interestingly, the Asu formation at LC-Asp-56 seems to block the generation of nearby *C*-terminal fragment ions (y_4_ and y_5_) and was not resolvable by variation of the low-energy CID conditions. This phenomena was already described in a recently published study on Asu formation at LC-Asp-30 of an IgG1 antibody [30].

**Figure 9:**
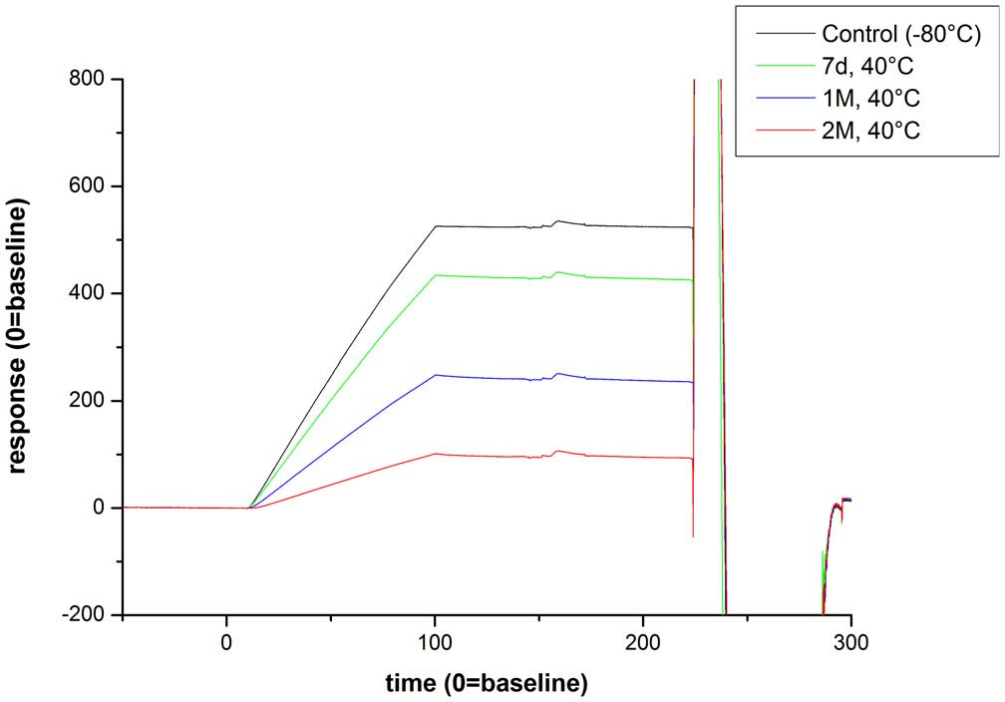
Analysis of Mab1 target binding by surface plasmon resonance. Biacore sensorgrams showing the target binding of non-stressed Mab1 material (stored at −80°C) and following elevated temperatures (storage at 40°C for 7 days, 1 month, and 2 month) at a flow rate of 100 µl/min.

Comparable with HER2, the elevated Asu formation in the CDR lead to a significant loss of target binding activity of Mab1 (Figure 9, Table 3).

## Discussion

For the identification of antibody CQAs derived from Asn deamidation and Asp isomerization events, a test system was applied employing incubation at elevated temperatures combined with proteolytic peptide mapping and quantitative UPLC-MS. The application of the histidine/HCl buffer system at pH 6.0 resulted in a significant reduction of method-induced deamidation events and the stabilization of Asu. For the recombinant IgG1 HER2 only 5 out of 14 LysC peptides containing Asn residues were found deamidated at quantifiable levels. Moreover, by applying UPLC-MS it was possible to monitor all relevant deamidated or isomerized LysC/tryptic peptide species. Although LysC/trypsin has optimal operating pH of about 8 and LysC/trypsin in this study was applied to pH 6.0, we obtained almost complete sequence coverages of ≥98% for HER2 and MAb1 by LC-MS and routinely find similar sequence coverages for other mAbs using Procedure B (data not shown). Also the reducing power of DTT is optimal at pH>7. In this study, we exclusively used DTT at pH 6.0 without the finding of significant di-peptide levels caused by incomplete sample reduction. It should be noted that ionization efficiencies of peptides can differ significantly depending on their amino acid contents. Increasing hydrophobicity usually leads to an enhanced formation of ionic species. On the other hand also variation of the pI value of peptides also alters the ionization efficiency. Consequently, different ionization efficiencies have to be taken into account when discussing and interpreting the data obtained from the presented experiments. However, the detected levels of deamidated or isomerized LysC/tryptic peptide species do interpret the data achieved by cation exchange chromatography, ESI-MS of reduced antibody, and target binding analysis suggesting that the depicted deamidation and isomerization content is not significantly overestimated for the inspected peptide species.

The application of elevated temperature stress (25°C and 40°C) for the CQA assessment resulted in the identification of LC-Asn-30, HC-Asn-55, and HC-Asp-102 as potential HER2 degradation hot-spots. These results are in agreement with a previous study on HER2 charge variants [12], in which LC-iso-Asp-30 was found as a predominant degradation product of LC-Asn-30 following incubation at 30°C for 90 days and using a conventional digestion procedure at pH 7.5. However, we found a significant accumulation of LC-Asu-30 at both temperature conditions. This again demonstrates that proteolytic peptide mapping with procedure B stabilizes the Asu content by preventing method-induced conversion of Asu to Asp and iso-Asp at peptide level. This conclusion is supported by a recently published study on Asu formation at LC-Asp-30 of an IgG1 antibody where the Asu conversion to iso-Asp was minimized by a tryptic digestion at pH 7 for 1 h [30].

Chelius *et al.* identified HC-Asn-(387, 392, 393) as most susceptible deamidation motive in the conserved region of recombinant IgG1s [16]. However, for the HC-Asp-283 (features a Gly-Asp motive like HC-Asp-102) and HC-Asn-(387, 392, 393) of HER2 no significant alterations in Asu accumulation and only a moderate increase of Asp and iso-Asp formation was observed following incubation at 40°C for 2 month. In conclusion, the level of degradation was around 5 to 10 times lower than the detected levels at LC-Asn-30 and HC-Asp-102.

The described approach was employed to identify CQAs for the Mab1 IgG1. Mab1 shares with HER2 the LC-Asn-30, offers a HC-Asp-99/Gly-100/Asp-101 motive, and exhibits a novel LC-Asp-56/Gly-57 sequence in the CDR 2. In contrast to HER2, only minimal Asu accumulation and deamidation at Mab1 LC-Asn-30 was verified, suggesting that the differences in neighboring amino acid residues administrate a protective effect. Contrary to HER2, we found a distinct Asu but only a moderate iso-Asp formation of Mab1 HC-Asp-99/Asp-101. Nevertheless, the elevated Asu formation in the CDR 3 also led to a significant loss of target binding activity. In addition, significant Asu and iso-Asp formation of Mab1 LC-Asp-56 was demonstrated. Since Asu/iso-Asp generation at LC-Asp-56 coexists with higher levels of Asu/iso-Asp formation at HC-Asp-99/Asp-101, the impact of LC-Asp-56 degradation on bioactivity cannot be unambiguously evaluated. However, the Asu/iso-Asp formation at LC-Asp-56 is significantly elevated when compared to the conserved HC-Asn-(389, 394, 395) motive. The criticality of Asu formation at HC-Asn-55 for target binding activity was described in a previous study [14]. Thus, Asu/iso-Asp formation at HC-Asp-99/Asp-101 and LC-Asp-56 can be regarded as Mab1 CQAs.

In summary, the application of elevated temperatures combined with LysC/tryptic peptide mapping at pH 6.0 and quantitative UPLC-MS was adequate to identify and assess potential sites for antibody Asp isomerization and Asn deamidation. Accordingly, the evaluation of fermentation, purification, formulation, and storage conditions on antibody Asn deamidation and Asp isomerization can be conducted by monitoring susceptible marker peptides located in the CDRs of recombinant IgG1 antibodies.

## Materials and Methods

### Induction of Asn deamidation and Asp isomerization using accelerated degradation conditions

The recombinant IgG1 antibodies HER2 and Mab1 were expressed in a Chinese hamster ovary cell system. Both antibodies were manufactured at Roche Diagnostics, Penzberg, Germany using standard cell culture and purification technology. HER2 was formulated at a concentration of 25 mg/mL in a His-HCl buffer system (60 mM) at pH 6.0. Mab1 was formulated at a concentration of 10 mg/mL in a His-HCl buffer system (20 mM) at pH 6.0. To induce antibody Asn deamidation and Asp isomerization, the recombinant IgG1 antibodies HER2 and MAB1 were exposed to elevated temperatures (25°C and 40°C) for 7 days, 1 month, or 2 month.

### Proteolytic digest (Procedure B)

For the detection and quantification of Asn deamidation and Asp isomerization at peptide level, HER2 and Mab1 were denatured in 0.2 M His-HCl, 8 M Gua-HCl, pH 6.0 by diluting 350 µg of mAb in a total volume of 300 µL. For reduction, 10 µl of 0.1 g/mL dithiothreitol was added followed by incubation at 50°C for 1 hour. As a next step, the buffer solution was exchanged to a digestion buffer (0.02 M His-HCl, pH 6.0) using a NAP5®-gel filtration column (GE Healthcare, Buckinghamshire, UK). Subsequently, the NAP5®-eluate (500 µL) was mixed with 10 µL of a 0.25 mg/mL trypsin solution (Trypsin Proteomics grade, Roche, Penzberg, Germany) in 10 mM HCl or with 10 µl of a 0.25 mg/mL Lys-C solution (Endoproteinase Lys-C Sequenzing Grade, Roche, Penzberg, Germany) in water and incubated at 37°C for 18 (Procedure B). Procedure A sample preparation was carried out according to the method described by Hensel *et al.* [31].

### Analysis of proteolytic peptides by liquid-chromatography mass-spectrometry (LC-MS)

The tryptic peptide mixture was separated by RP-UPLC (ACQUITY, Waters, Manchester, UK) on a C18 column (BEH C18 1,7 µm 2,1×150 mm; Waters, Manchester, UK) and the eluate online analyzed with a LTQ Orbitrap Velos electrospray mass spectrometer (Thermo, Waltham, MA, US). The mobile phases consisted of 0.1% formic acid in water (solvent A) and 0.1% formic acid in acetonitrile (solvent B). The chromatography was carried out using a gradient from 1 to 35% solvent B in 45 min and finally from 35 to 80% solvent B in 3 min using a flow rate of 300 µL/min. UV absorption was measured at a wavelength of 220 nm. 3.5 µg digested protein was applied. UPLC-system and mass spectrometer were connected by PEEK-capillary tubes. Data acquisition was controlled by XCalibure software (Thermo, Waltham, MA). Parameters for MS detection were adjusted according to general experience available from peptide analysis of recombinant antibodies.

### Data analysis for the quantification of deamidation/isomerization levels

Peptides of interest were identified manually by searching their *m/z*-values within the experimental mass spectrum. For the quantification, specific ion current (SIC) chromatograms of peptides of interest were generated on the basis of their monoisotopic mass for all detectable charge states using GRAMS AI software (Thermo Fisher Scientific, Dreieich, Germany). Relative amounts of Asn deamidation and Asp isomerization were calculated by manual integration of modified and unmodified peptide peaks. For example, the relative content of LC-Asu-30 (Tables 1, 2, 3) is calculated by the integrated SIC chromatogram area of LC-Asu-30 divided by the sum area of all LC-Asn-30 peptide species (LC-Asn-30, LC-Asu-30, LC-Asp-30, and LC-isoAsp-30).

### Identification of peptides by LC-MS/MS

MS/MS experiments were performed on-line on a Q-TOF SYNAPT G2 instrument (Waters, Manchester, UK) using the described chromatographic system. Peptides of interest were isolated on the basis of their mass and charge state and fragmentation induced by low-energy CID using helium as collision gas. The collision energy was adjusted according to stability and mass of the parent ion. Data acquisition was controlled by MassLynx software (Waters, Manchester, UK) using a manual acquisition mode. MS/MS-data were analyzed manually using MassLynx software for mass detection and data interpretation.

### NanoESI-QTOF MS of HER2 and Mab1

Mass spectrometric analysis of reduced antibody light and heavy chains was performed on a NanoESI-QTOF system (Q-TOF Ultima, Waters, Manchester, UK). The spectra were recorded in the positive ion mode using acetonitrile/water/formic acid (79/20/1, v/v/v) as solvent.

### Cation exchange chromatography (CEC)

Ion exchange chromatography was performed to monitor Mab1 charge variants using a ProPac WCX-10 Analytical cation exchange column (4.0×250 mm; Dionex Softron GmbH, Germering, Germany). A step gradient using 20 mM MES, pH 6.2 as solvent A and 20 mM MES, 750 mM NaCl, pH 6.2 as solvent B at 1.0 mL/min was applied. Chromatographic separation was executed on an Ultimate3000 HPLC-system equipped with UV detection at 280 nm. 50 µg mAb pre-treated with Carboxypeptidase B (0.5 mg/mL) for 30 min at 37°C was injected for the chromatographic analysis.

### Size exclusion chromatography (SEC)

Size exclusion chromatography was carried out using a TSK-Gel G3000SWXL column (7.8×300 mm, 5 µm particle size; Tosoh Bioscience, Amsterdam, Netherlands). An isocratic elution using 200 mM KH_2_PO_4_, 250 mM KCl, pH 7.0 at 0.5 mL/min as solvent was used for chromatographic separation on an Ultimate3000 HPLC-system equipped with UV detection at 280 nm. 150 µg of mAb was injected for the chromatographic analysi and data acquisition was controlled by Chromeleon software (Dionex Softron GmbH, Germering, Germany). Relative quantification was performed by manual integration and comparison of peak areas.

### Analysis of target binding by surface plasmon resonance (SPR)

The interaction between the stressed or non-stressed mAb and the specific target protein was measured by surface plasmon resonance using a Biacore T100 instrument (GE Healthcare Bioscience, Uppsala, Sweden). To evaluate MAb interaction to its specific target, the assay type “Calibration-free Concentration analysis” (BiaEvaluation Software, GE Healthcare Bioscience, Uppsala, Sweden) was performed. The specific target protein was immobilized onto a Biacore CM5-biosensor chip (GE Healthcare Bioscience, Uppsala, Sweden) via amine coupling to reach maximum coupling density. The assay was carried out at room temperature with HBS-P-buffer (GE Healthcare Bioscience) as running and dilution buffer. 20 nM of native or stressed MAb samples were injected at a flow rate of 5 µL/min and 100 µL/min at room temperature, respectively. Association time was 90 s, dissociation phase took 30 s. Regeneration of the chip surface was reached by a short injection of 4 M MgCl_2_. Evaluation of SPR-data was performed by comparison of the biological active amount of an non-stressed sample with stressed samples. Biological activity of non-stressed samples was set to 100%.
